# Establishment of genetic tools for genomic DNA engineering of *Halomonas* sp. KM-1, a bacterium with potential for biochemical production

**DOI:** 10.1186/s12934-022-01797-2

**Published:** 2022-06-20

**Authors:** Ayaka Tsuji, Yasuko Takei, Yoshinao Azuma

**Affiliations:** Graduate School of Biology-Oriented Science and Technology, Kindai University, Wakayama, Japan

**Keywords:** Expression vector, CRISPR-Cas9, Hemolysin coregulated protein, *pyrF*, Polyhydroxybutyrate

## Abstract

*Halomonas* species are halophilic and alkaliphilic bacteria, which exhibit potential for industrial production of a variety of chemicals, such as polyhydroxyalkanoates and ectoine, by fermentation because of their favorable characteristics, including high-density culturing capacity and low risk of contamination. However, genetic tools to modify the metabolism of *Halomonas* for suitable fermentation performance are limited. In this study, we developed two independent basic vectors for *Halomonas*, named pUCpHAw and pHA1AT_32, consisting of *ori* regions from two plasmids isolated from *Halomonas* sp. A020, and chloramphenicol- and tetracycline-resistant genes as cloning markers, respectively. These vectors can independently transform and co-transform the *Halomonas* sp. KM-1 (KM-1). A protein that was highly and constitutively accumulated was identified as a hemolysin coregulated protein (Hcp) based on proteome analysis of KM-1. Using the *hcp* promoter, various genes, such as *phaA* and EGFP, were highly expressed. To establish a gene disruption system, the *Streptococcus pyogenes cas9* gene and guide RNA for the *pyrF* gene, a yeast *URA3* homologue, were expressed in pUCpHAw and pHA1AT_32, respectively. As a result, gene disruption mutants were isolated based on phenotypes, 5-fluoroorotic acid resistance, and uracil auxotrophy. A combination of KM-1 and these vectors could be a suitable platform for industrial chemical and protein production.

**Supplementary Information:**

The online version contains supplementary material available at 10.1186/s12934-022-01797-2.

## Introduction

*Halomonas* species are gram-negative bacteria that are moderately halophilic, alkaliphilic, and aerobic, which are often isolated from salty environments such as salterns [1] and hypersaline lakes [2]. There has been 113 published and registered strains in the genus *Halomonas* [3]. One of the most significant characteristics of *Halomonas* strains is the diversity of biochemical production, such as ectoine (1,4,5,6-tetrahydro-2-methyl-4-pyrimidine carboxylic acid) by *Halomonas elongata* [4], polyhydroxybutyrate (PHB) by *Halomonas bluephagenesis* TD01 [5], and (*R*)-3-hydroxybutyrate (3HB) by *Halomonas* sp. KM-1 (KM-1) [6]. *Halomonas* species can grow in highly saline and alkaline media, which can be too severe for most environmental microbes to proliferate; thus, fermentation can be performed without time-consuming and costly sterilization of media. Another advantageous characteristic is a strong proliferating ability, such as cell growth to high turbidity values and utilization of various carbon sources, including waste glycerol [7]. Thus, biochemical industrial production using *Halomonas* is expected to save energy with a lower burden on the environment [8].

Intended for the industrial development of *Halomonas*, several genetic tools and methods have been developed, such as gene transfer by conjugation using a broad-host-range vector in *H. elongata* and *Halomonas subglaciescola* [9], an effective electroporation method for transformation using *Halomonas* sp. O-1 [10], and gene disruption and homologous recombination in *H. bluephagenesis* TD01 using CRISPR-Cas9 systems [11, 12]. To select mutants after gene knockin and disruption in yeast, *URA5*/*pyrE* and *URA3*/*pyrF* genes involved in pyrimidine synthesis have been utilized for positive selection together with 5-fluoroorotic acid (5-FOA) [13]. In *Halomonas*, *pyrF* was adopted as a *pyrF*-mediated gene disruption method in *Halomonas campaniensis* L21 [14] and used as a positive selection marker in a complementation-base expression system in a *pyrF*-deficient mutant of *H. bluephagenesis* TD01 [15]. The *pyrF* (and *pyrE*) gene appears to be a strong candidate for gene disruption and a positive selection marker in other *Halomonas* strains.

KM-1 was isolated as a bacterium that produces PHB under aerobic conditions from the culture of the cyanobacterium *Spirulina platensis* under high pH and salt conditions with 3% glycerol as a sole carbon source [7]. Interestingly, KM-1 was also shown to secrete organic acids into media such as 3HB under microaerobic conditions [6], and pyruvate and oxaloacetate under aerobic conditions [16, 17]. Thus, KM-1 has specific advantages for industrial fermentation applications [6, 16]. The draft genome sequence for KM-1 has been reported, and most of the genes and regulatory regions are available to enhance their abilities. In this study, we developed genetic tools to manipulate the metabolism of KM-1 using a variety of promoters and a gene disruption system utilizing the CRISPR-Cas9 system based on two independent shuttle vectors prepared from two plasmids identified in *Halomonas* sp. A020 [18].

## Materials and methods

### Bacterial strains and cultural conditions

*Escherichia coli* strain DH5α was used for gene cloning and vector construction in this study. *Halomonas* sp. KM-1 (FERM BP-10995) and its genome information (GenBank assembly accession GCA_000246875.2) were used to characterize the vectors and gene disruption [7, 19]. KM-1 was gifted by Dr. Kawata at the National Institute of Advanced Industrial Science and Technology. *Halomonas* sp. A020 (Accession No. AP022850) isolated from a Japanese pickled plum factory was used to isolate native plasmids [18]. The broad-host-range vector pBBR1MCS was gifted by Dr. Tsuda at Tohoku University.

LB medium (1% tryptone, 0.5% yeast extract, and 1% NaCl) was used to culture *E. coli*. *Halomonas* growth medium (HGM) (pH 8.0, 1% tryptone, 0.5% yeast extract, and 3.5% NaCl) was used for the electroporation method of *Halomonas* transformation [10]. SOT (pH 9.5, 3% (w/v) sucrose, 2.5% NaCl) and SOT plate (2% agar) media were used for the general culture of *Halomonas* species [7]. Liquid culturing of *Halomonas* in HGM and SOT medium was performed at 30 °C under agitation at 250 rpm and 33 °C under agitation at 200 rpm, respectively.

### Chemicals, enzymes, and molecular biology kits

5-FOA (Tokyo Chemical Industry, Tokyo, Japan), uracil and chloramphenicol (FUJIFILM, Tokyo, Japan), ampicillin (Sigma-Aldrich, St. Louis, MO, USA), tetracycline, isopropyl β-D-1-thiogalactopyranoside (IPTG), and a protease inhibitor cocktail (Nacalai Tesque, Kyoto, Japan) were used for mutant selection, gene expression, and protein analysis. DNA polymerase for polymerase chain reaction (PCR) and In-Fusion HD Cloning Kit were purchased from TaKaRa Bio Inc. (Shiga, Japan). DNA purification was performed using Spin Miniprep Kit for plasmid DNAs and Gentra Puregene Yeast/Bact. Kit for genomic DNAs (QIAGEN, Hilden, Germany). Guide-it Cas9 polyclonal antibody and goat anti-rabbit IgG horseradish peroxidase (HRP)-linked antibody were purchased from TaKaRa Bio Inc. and Agilent (P0448, Agilent, Santa Clara, CA, USA), respectively. Enhanced chemiluminescence (ECL) prime, 2-D Clean-Up Kit, and Immobiline DryStrips were purchased from Cytiva (Tokyo, Japan).

### Shuttle vector construction

**Fig. 1:**
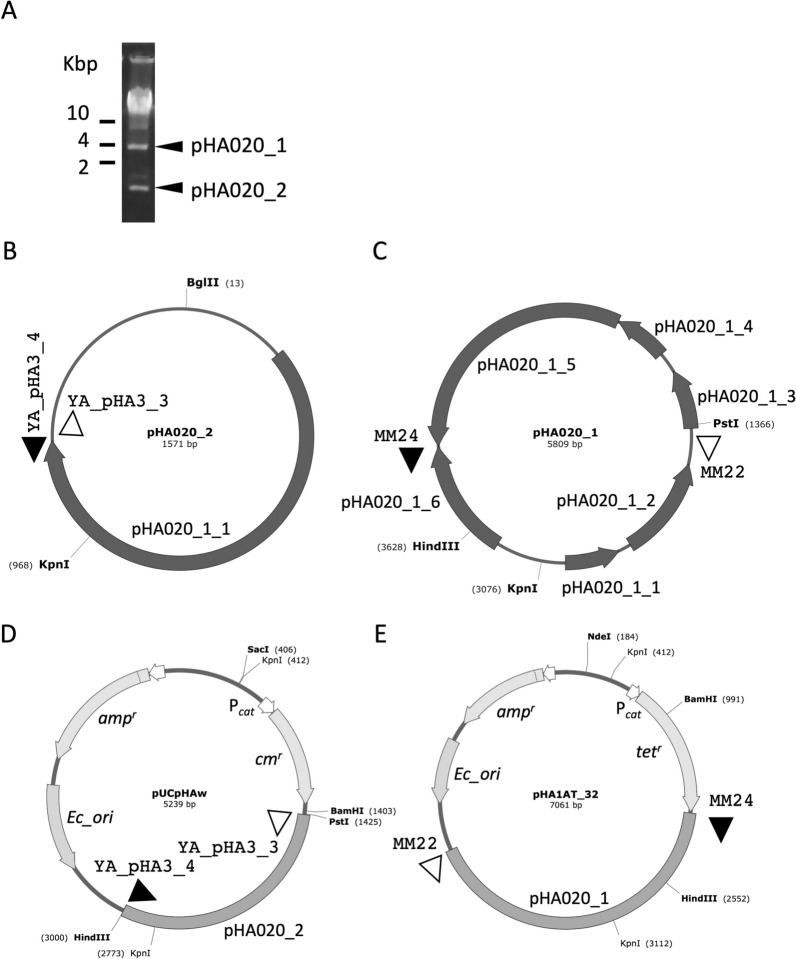
Construction of shuttle vectors. **A** Two plasmid DNAs were detected in the *Halomonas* sp. A020. **B**, **C** Large and small plasmids in A020 were named pHA020_1 and pHA020_2, respectively. Six and one genes were identified and annotated in pHA020_1 and pHA020_2, respectively. Annotations of the genes are listed in Table 1. Locations of primers to clone the predicted replication origins are indicated with black and white triangles. **D** Gene map of the first shuttle vector, pUCpHAw, 5239 bp in length. The *cm*^*r*^ and P_*cat*_ indicate chloramphenicol-resistant gene (*cm*^*r*^*/cat*) and its promoter from pG-KJE8, respectively. pHA020_2 shows the entire region of pHA020_2. *Ec_ori* and *amp*^*r*^ are *E. coli ori* and an ampicillin-resistant gene from pUC19, respectively. All vectors are listed in Table 2 and the detailed vector construction is shown in Additional file 1: Fig. S1. Black and white triangles indicate positions of primers corresponding to panel **B**. **E**) Gene map of the second shuttle vector, pHA1AT_32, 7061 bp in length. The *cm*^*r*^ and pHA020_2 in the pUCpHAw were substituted with a tetracycline-resistant gene (*tet*^*r*^) from YEp13 and the replication origin in the pHA020_1, respectively. The detailed vector construction is shown in Additional file 1: Fig. S2. Black and white triangles indicate positions of primers corresponding to panel **C**

Two independent *Halomonas*—*E. coli* shuttle vectors were established using *Halomonas* replication origins in the small plasmid pHA020_2 (Accession No. AP022852), and a large pHA020_1 (Accession No. AP022851) of *Halomonas* sp. A020 (Fig. 1, Table 1) [18]. One of the shuttle vectors, pUCpHAw (Fig. 1D), was constructed using In-Fusion cloning, as per the manufacturer’s instructions. Briefly, a DNA fragment containing a chloramphenicol resistant gene (*cm*^*r*^*/cat*) from pG-KJE8 was amplified by PCR with primers (YA_pChl1 and YA_pChl2, DNA sequences in Additional file 1: Table S1) and cloned into the *Sma*I site of pUC19 using In-Fusion cloning, followed by cloning of the whole pHA020_2 DNA, amplified by PCR with primers (YA_pHA3_3, YA_pHA3_4), into an *Sph*I site of pUC19 (Additional file 1: Fig. S1). The other, pHA1AT_32 (Fig. 1E), was constructed from pUCpHAw by substitution of the origin and selection marker genes. First, the pHA020_2 region in pUCpHAw was removed by PCR with primers (MM26 and MM27) and exchanged with the origin region of pHA020_1 amplified by PCR with primers (MM22 and MM24). The *cm*^*r*^ gene was then removed by PCR with primers (MM28 and MM29) and substituted with a tetracycline-resistant gene (*tet*^*r*^) of YEp13 amplified by PCR with primers (MM12 and MM31) (Additional file 1: Fig. S2). Plasmids and constructed vectors in this study are listed in Table 2, and the primers are listed in Additional file 1: Table S1. Plasmid maps were created using SnapGene Viewer 6.0.2 available at snapgene.com (Insightful Science). The DNA sequences of pUCpHAw and pHA1AT_32 were attached as Additional files 2 and 3, respectively.

**Table 1:** Genes on the plasmids, pHA020_1 and pHA020_2

Gene	Annotation
pHA020_1_1	Hypothetical protein
pHA020_1_2	IS5 family transposase
pHA020_1_3	Hypothetical protein
pHA020_1_4	MobC family plasmid mobilization relaxosome protein
pHA020_1_5	Rho containing hypothetical protein
pHA020_1_6	Rep3 containing replication initiation protein
pHA020_2_1	Phage replicon protein

**Table 2:** Vectors used in this study

Name	Description	References
Vector		
pBBR1MCS	A broad-host-range vector, gifted from Prof. Tsuda, Grad. Sch. of Life Science, Tohoku Univ. (GenBank U02374)	Kovach et al. 1994 [42]
pHA020_2	A smaller plasmid in *Halomonas* sp. A020 (Accession No. AP022852)	Tsuji et al. 2021 [18]
pHA020_1	A larger plasmid in *Halomonas* sp. A020 (Accession No. AP022851)	Tsuji et al. 2021 [18]
pUC19	A cloning vector (GenBank M77789)	Yanisch-Perron et al. 1985 [43]
pG-KJE8	A chaperone plasmid containing *cm*^*r*^, purchased from Clontech (TaKaRa Bio)	Nishihara et al. 2000 [44]
YEp13	A YE type *Saccharomyces cerevisiae*—*E. coli* shuttle vector containing *tet*^*r*^ (GenBank U03498)	Broach 1979 [45]
pEGFP	A bacterial expression vector containing EGFP tag, purchased from Clontech (TaKaRa Bio)	Inouye et al. 1994 [46]
pTrc99a	A bacterial expression vector with *lacI*^*q*^ regulated *trc* promoter (GenBank M22744)	Amann et al. 1988 [47]
pwtCas9-bacteria	A Tet-inducible expression vector of *Streptococcus pyogenes cas9* gene, purchased from Addgene (Addgene plasmid # 44,250)	Qi et al. 2013 [48]
pgRNA-bacteria	An expression vector of Cas9 guide RNA for bacterial gene disruption. purchased from Addgene (Addgene plasmid # 44,251)	Qi et al. 2013 [48]
KM-1 vector		
pUCpHAw	pUC19 derivate including whole pHA020_2 and *cat*/*cm*^*r*^	This work
pHA1AT_32	pUC19 derivate including an origin region of pHA020_1 and *tet*^r^ under a *cat* promoter	This work
Gene expression vector		
pUCpHAw_EGFP	pUCpHAw derivate including EGFP gene at a *Pst*I site of the pUCpHAw	This work
pUCpHAw_Phcp_EGFP	pUCpHAw_EGFP derivate including a promoter region of *hcp* gene	This work
pUCpHAw_Pphasin_EGFP	pUCpHAw_EGFP derivate including a promoter region of *phasin* gene	This work
pUCpHAw_Ptrc_EGFP	pUCpHAw_EGFP derivate including *trc* promoter with *lacI*^*q*^ of pTrc99a	This work
pCmHAw_Phcp_zwf	pUCpHAw_Phcp_EGFP derivate substituted EGFP gene to *zwf* gene	This work
pCmHAw_Phcp_phaA	pUCpHAw_Phcp_EGFP derivate substituted EGFP gene to *phaA* gene	This work
pCmHAw_Phcp_tesB	pUCpHAw_Phcp_EGFP derivate substituted EGFP gene to *tesB* gene	This work
pCmHAw_Phcp_pyrE	pUCpHAw_Phcp_EGFP derivate substituted EGFP gene to *pyrE* gene	This work
pCmHAw_Phcp_pyrF	pUCpHAw_Phcp_EGFP derivate substituted EGFP gene to *pyrF* gene	This work
Gene disruption vector		
pTHA(Cas9)	pUCpHAw derivate including a DNA fragment containing *lacI*^*q*^ and *cas9* under a *trc* promoter	This work
pgRNAHA_pyrF	pHA1AT_31 derivate including a guide RNA for *pyrF* gene	This work
pgRNAHA	pHA1AT_32 derivate including an original guide RNA of pgRNA-bacteria	This work

### Expression vector construction

A vector for gene expression, pUCpHAw_EGFP, was constructed using enhanced green fluorescence protein (EGFP) as a reporter gene for promoter analysis. Briefly, an EGFP coding region amplified by PCR using pEGFP (TaKaRa Bio Inc.) and primers (AT031, AT044) was cloned into the *Pst*I site of pUCpHAw by In-Fusion cloning (Additional file 1: Fig. S3). Three promoter regions, upstream regions of *hcp* and *phasin* genes of KM-1 and *trc* promoter with *lacI*^*q*^ of pTrc99a vector, were amplified by PCR using primers (AT098 and AT099), (AT143 and AT144), and (AT141 and AT142), respectively (Additional file 1: Fig. S3). These DNA fragments were assembled with a DNA fragment of pUCpHAw_EGFP amplified by PCR with primers (YA_pChl2, AT097), resulting in three vectors: pUCpHAw_Phcp_EGFP, pUCpHAw_Pphasin_EGFP, and pUCpHAw_lacI^q^_Ptrc_EGFP.

Ampicillin resistance gene (*amp*^*r*^) in pUCpHAw_Phcp_EGFP was removed by PCR with primers (MM05 and MM06), resulting in pCmHAw_Phcp_EGFP. Seven KM-1 genes, including *zwf*, *phaA*, and *tesB*, were cloned by exchange with EGFP in pCmHAw_Phcp_EGFP (Additional file 1: Fig. S4). The DNA sequence of pUCpHAw_EGFP was attached as Additional file 4.

### Vector construction for gene disruption

The pTHA(Cas9) vector, which expresses the *S. pyogenes cas9* gene in KM-1, was constructed with pUCpHAw and pwtCas9-bacteria (Addgene plasmid #44,250) (Additional file 1: Fig. S5). First, the *cas9* gene fragment was amplified by PCR using pwtCas9-bacteria and primers (YA_cas9a, YA_cas9b), and assembled with a *Nco*I-*Hind*III fragment of the pTrc99a vector using In-Fusion cloning. The DNA fragment containing *lacI*^*q*^, *trc* promoter, and *cas9* was amplified by PCR with primers (YA_cas9c, YA_cas9d) and assembled with a *Sac*I-*Hind*III fragment of pUCpHAw.

The pgRNAHA_pyrF vector to express a guide RNA for Cas9 protein was constructed with pHA1AT_32, pgRNA-bacteria (Addgene plasmid #44,251), and a base-pairing region with *pyrF* gene of KM-1 (Additional file 1: Fig. S6). A 20 bp DNA fragment in *pyrF* adjacent to a PAM sequence was cloned into pgRNA-bacteria by PCR with primers (YT01 and YT02) and self-ligation. After *Bam*HI digestion of the vector, the DNA fragment was assembled with pHA1AT_32 amplified by PCR with primers (AT170 and AT171) by In-Fusion, resulting in pgRNAHA_pyrF. To construct a control vector, pgRNAHA, a DNA fragment of pgRNA-bacteria digested with *Bam*HI was assembled with the pHA1AT_32 fragment in the same way as gRNAHA_pyrF construction.

To complement the *pyrF* gene mutation, two vectors, pCmHAw_Phcp_pyrF and pCmHAw_Phcp_pyrE, were prepared by replacing the EGFP gene in pCmHAw_Phcp_EGFP with KM-1 *pyrF* and *pyrE* genes, respectively (Additional file 1: Fig. S7). The pCmHAw_Phcp_EGFP vector, *pyrF*, and *pyrE* were amplified by PCR with primers (MM54 and MM55), (MM80 and MM81), and (MM84 and MM85), respectively, and assembled using In-Fusion. The DNA sequences of pgRNAHA_pyrF and pTHA(Cas9) were attached as Additional files 5 and 6, respectively.

### Electroporation method of KM-1 transformation

Electrocompetent KM-1 cells were prepared using a previously reported method [10]. Briefly, KM-1 was cultured in HGM at 30 °C under agitation at 250 rpm and collected at an OD_600_ of 0.5, by centrifugation for 10 min at 5000×*g* at 25 °C. After washing the cells twice with 300 mM sucrose solution, the cells were resuspended in 300 mM sucrose and immediately frozen in liquid nitrogen. Transformation of KM-1 was conducted in 0.2 cm gap parallel electrode cuvettes using an Eppendorf Eporator (Eppendorf, Hamburg, Germany) at 2100 V. Cells were resuspended in 1 mL of HGM and incubated for 3–4.5 h at 30 °C with agitation at 250 rpm. Transformed clones were selected on HGM plates containing 1.5% agar, 2.5 µg/mL chloramphenicol or 5 µg/mL tetracycline as final concentrations. For the secondary transformation, HGM plates were prepared with 2.5 µg/mL chloramphenicol and 3 µg/mL tetracycline as final concentrations.

### Microscope analysis

Bacterial cells were fixed in 4% paraformaldehyde phosphate buffer for 5 min at room temperature and washed with 1 × Dulbecco’s Phosphate Buffered Saline (pH 7.2, D1408, Sigma-Aldrich). Cells were observed using an inverted fluorescence microscope (BZ-X800, KEYENCE, Osaka, Japan).

### Protein analysis

For western blotting of Cas9 protein, sodium dodecyl sulfate–polyacrylamide gel electrophoresis (SDS-PAGE) was performed using a 7.5% acrylamide gel, and proteins were blotted onto a PVDF membrane. Immuno-detection was performed using Cas9 antibody and anti-rabbit IgG HRP-linked antibody. Finally, signals were detected using ECL prime (Cytiva, Tokyo, Japan) and LuminoGraph II (WSE-6200H, ATTO, Tokyo, Japan).

For 2D-PAGE analysis of protein accumulation, KM-1 was incubated for 24 h in SOT medium supplemented with 10% sucrose at 33 °C under agitation at 200 rpm. Bacterial cells were collected and disrupted in 2D-PAGE sample buffer (8.3 M urea, 2% w/v CHAPS, a protease inhibitor cocktail) using an ultrasonic homogenizer (VP-050, Taitec, Nagoya, Japan). After centrifugation to remove the insoluble fraction, proteins were purified using 2-D Clean-Up Kit (Cytiva, Tokyo, Japan) and applied onto an Immobiline DryStrip (pH 4–7, 13 cm, Cytiva, Tokyo, Japan). Isoelectric focusing was conducted using PowerPhoreStar Pro3900 and CoolPhoreStar IPG-IEF Type-PX (Anatech, Tokyo, Japan). After equilibration with the equilibration buffer (6 M urea, 2% SDS, 35% v/v glycerol, 50 mM Tris–HCl [pH 6.8], BPB), the gel strip was subjected to SDS-PAGE using a 12.5% polyacrylamide gel.

### Gene disruption

KM-1 was transformed using the pTHA(Cas9) vector, and the accumulation of Cas9 protein by supplementation with IPTG was tested using western blotting. The KM-1 with pTHA(Cas9) was transformed using the pgRNAHA_pyrF vector. The strain harboring both of pTHA(Cas9) and pgRNAHA_pyrF vectors was cultured in HGM supplemented with 3 µg/mL chloramphenicol, 5 µg/mL tetracycline, and 10 µg/mL uracil at 30 °C with constant agitation at 250 rpm. At approximately 0.5 OD_600_, IPTG was added at a final concentration of 1 mM and incubated for 14 to 40 h. The cells in 0.2–1.0 mL of culture were spread on 3% sucrose SOT plates with 3 µg/mL chloramphenicol, 10 µg/mL uracil, and 0.5 mg/mL 5-FOA and incubated at 33 °C for 48 h. The strains showing 5-FOA resistance and uracil auxotrophy were selected, and DNA fragments including the *pyrF* gene were sequenced to identify the mutations.

### Gene identification and bioinformatics

Partial genomic DNA sequencing of KM-1 was conducted using a whole-genome shotgun strategy, using a method described previously [20]. Briefly, 3.8 μg of genomic DNA extracted from KM-1 were fragmented and isolated using the NEBNext Ultra II DNA Library Prep Kit (Illumina, San Diego, CA, USA), and a total of 9,413,6344 reads (paired-end 150 bp) were generated using HiSeq (Illumina). Short DNA reads were assembled using CLC Genomics Workbench 20 software (QIAGEN), resulting in a contig length of 70,092 bp in N50 and a total of 4,601,538 bp in 50 longest contigs (average of coverage: × 245). Gene analysis was performed using CLC Genomics Workbench to find out the genes encoding the N-terminal amino acid sequences acquired in the protein analysis.

## Results

### Construction of shuttle vectors for KM-1

The high-efficiency transformation of *Halomonas* sp. O-1 using the electroporation method was previously reported using a broad-host-range vector, pBBR1MCS [10], but the transformation efficiency of *Halomonas* sp. KM-1 by the vector was 100 times lower than that reported in a previous report (Additional file 1: Table S2). We previously reported that the de novo isolated *Halomonas* sp. A020 included two plasmids, pHA020_1 and pHA020_2 (Fig. 1A–C) [18]. These plasmids were predicted to contain six and one genes, respectively (Table 1), and the replication origins of the plasmids were adapted to develop shuttle vectors for KM-1.

The first vector pUCpHAw was constructed with the whole region of the pHA020_2 plasmid and *cm*^*r*^*/cat* as a selection marker (Fig. 1D, Additional file 1: Fig. S1). To construct the second vector, the pHA020_2 region in pUCpHAw was substituted with several fragments of pHA020_1, and the longest non-coding region in pCmHA1_3 was found to function as a replication origin in KM-1. The second vector, pHA1AT_32, was constructed using the pCmHA1_3 and *tet*^*r*^ genes (Fig. 1E, Additional file 1: Fig. S2). Transformation of KM-1 using the electroporation method with pUCpHAw and pHA1AT_32 was performed on HGM supplemented with 2.5 µg/mL chloramphenicol and 5 µg/mL tetracycline, respectively. Transformation efficiencies of KM-1 using pUCpHAw and pHA1AT_32 vectors were both approximately 10^2^–10^3^ CFU (colony formation units)/µg DNA and approximately 10 times higher than that of pBBR1MCS (Additional file 1: Table S2).

### Promoter selection for expression vectors

**Fig. 2:**
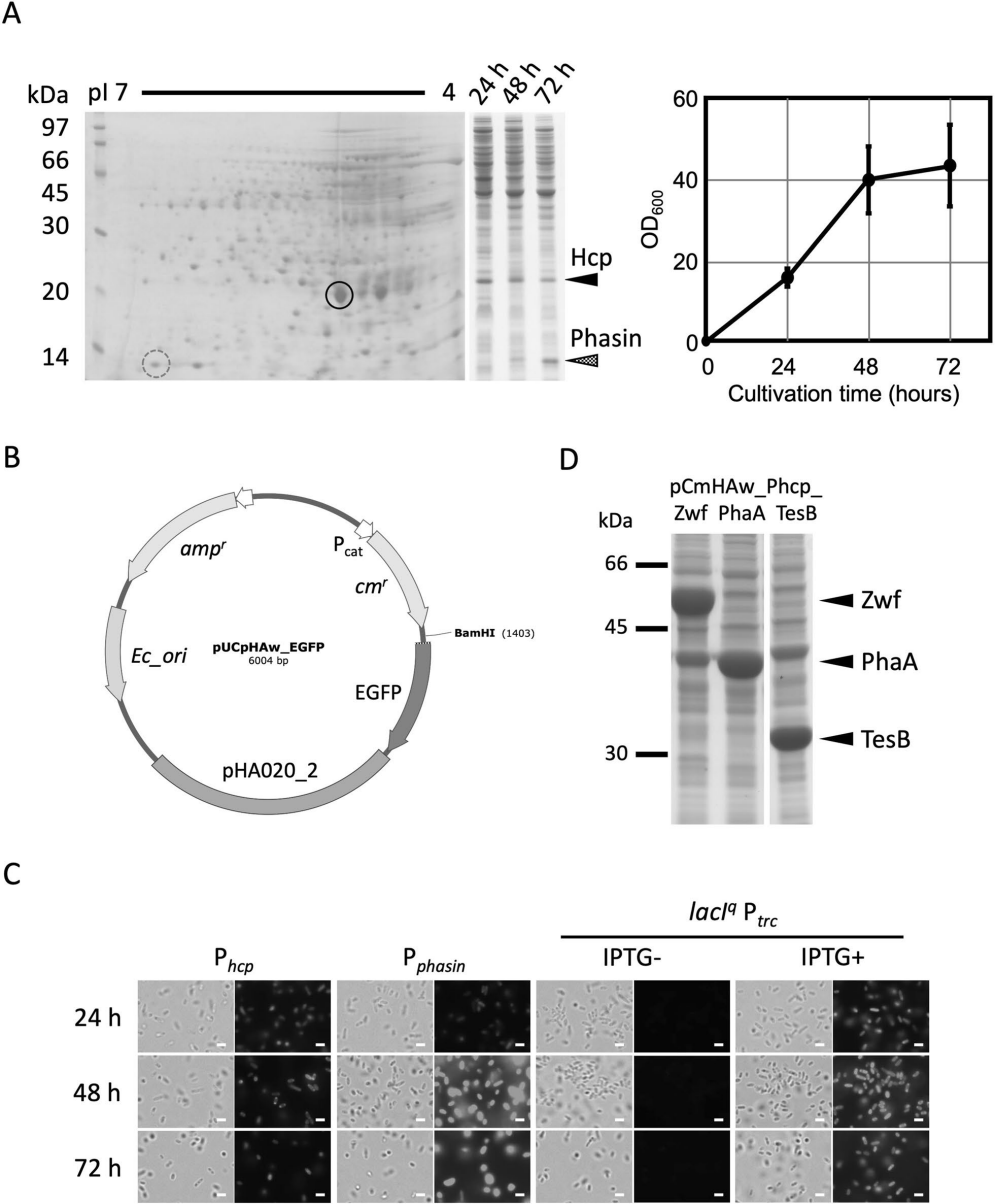
Promoter characterization for expression vectors. **A** Soluble and total proteins of KM-1 were analyzed using 2D (a left panel) and 1D (a middle panel) gel electrophoresis. For the 1D analysis cells were collected at 24-, 48-, and 72-h incubation in SOT medium supplemented with 10% sucrose at 33 ℃ under agitation at 200 rpm (a right panel). Cells collected at 24-h incubation were used for 2D analysis. A spot at pI 5 and 20 kDa, indicated in a solid circle, is identified as an Hcp family type VI secretion system effector (Accession No. LC677173). The other spot at pI 7 and 14 kDa in a dotted circle is identified as a polyhydroxyalkanoate-associated protein (Phasin, Accession No. LC677174). **B** pUCpHAw_EGFP vector was constructed (Additional file 1: Fig. S3) to evaluate promoters of *hcp* and *phasin* genes and *trc* promoter. The three promoters, P_*hcp*_*,* P_*phasin*_, and P_*trc*_ were cloned upstream of the EGFP gene. P_*trc*_ was cloned together with an *E. coli lacI*^*q*^ gene. **C** Using EGFP as a reporter, expression levels of *hcp* and *phasin* promoters were investigated in KM-1 as well as *E. coli trc* promoter regulated by *lacI*^*q*^. Cells were cultured in SOT medium supplemented with 10% sucrose and 5 µg/mL chloramphenicol at 33 ℃ under agitation at 200 rpm. Cultivation times (in hours) are shown on the left. IPTG-induction was started at 10 h incubation with a final concentration of 1 mM, and cells were observed at 14-, 38-, and 62-h after IPTG-induction. Left and right photos in each panel are visual and fluorescent observations in the same fields. Scale bars show 1 µm. **D** Seven genes including *zwf*, *phaA*, and *tesB* were exchanged with EGFP in the pCmHAw_Phcp_EGFP vector. KM-1 harboring the vectors were cultured in SOT medium supplemented with 10% sucrose and 2.5 µg/mL chloramphenicol at 33 ℃ under agitation at 200 rpm and collected at 48-h cultivation. Expected positions of gene products, Zwf (57 kDa, sequence ID WP_010627120.1), PhaA (41 kDa, WP_010626348.1), and TesB (30 kDa, WP_010629752.1), are indicated with black arrows

To identify highly expressed and regulated gene promoters, proteome analysis of KM-1 was conducted using cells harvested at different growth stages (Fig. 2A). Based on the results of 1D- and 2D-PAGE, one highly accumulated protein was detected at pI 5 and 20 kDa in the exponential stage between 24 and 48 h, and the other spot at pI 7 and 14 kDa was detected as highly accumulated protein in the stationary phase at 72 h (Fig. 2A). Based on the N-terminal amino acid sequence of the 14 kDa protein, the gene encoding the protein was found in a contig of the KM-1 genome (BAEU01000063.1) (Additional file 1: Table S3). The protein (LC677174) was presumed as a polyhydroxyalkanoate-associated protein and classified into the Phasin 2 family, which is widely conserved among other *Halomonas* species (Additional file 1: Fig. S8A). The N-terminal amino acid sequencing of the 20 kDa protein revealed that it might be a part of a hemolysin coregulated protein (Hcp) family type VI secretion system (T6SS) effector, but no gene coding the amino acid sequence was identified in the draft genome of KM-1 (Additional file 1: Table S3). Thus, sequencing of KM-1 genomic DNA was performed using Illumina shotgun sequencing. An open reading frame encoding the N-terminal amino acid of Hcp was found in a contig and the whole amino acid sequence of Hcp predicted from the gene was highly similar to those of Hcp of other *Halomonas* and some γ-proteobacteria species (Additional file 1: Fig. S8B).

Both of the *hcp* and *phasin* genes were predicted as a monocistronic gene or the first gene in the operon based on the distance from a flanking gene. Thus, upstream non-coding regions adjacent to the genes were cloned upstream of the EGFP gene in the reporter vector pUCpHAw_EGFP (Fig. 2B, Additional file 1: Fig. S3), resulting in pUCpHAw_Phcp_EGFP and pUCpHAw_Pphasin_EGFP, respectively. In addition, to evaluate whether the IPTG induction system of *E. coli* is suitable in KM-1, the *trc* promoter region was cloned into pUCpHAw_EGFP with the operator gene, *lacI*^*q*^, from the pTrc99a vector, resulting in pUCpHAw_lacI^q^_Ptrc_EGFP (Additional file 1: Fig. S3). After the transformation of KM-1 with each vector, EGFP expression was observed using fluorescence microscopy (Fig. 2C). The *hcp* promoter constitutively exhibited EGFP expression, while the *phasin* promoter showed high expression of EGFP in the stable phase at 48–72 h, likely due to its natural expression. EGFP fluorescence under the *trc* promoter was strongly induced by the addition of IPTG (Fig. 2C). The *hcp* promoter was used to express seven more KM-1 endogenous genes (Additional file 1: Fig. S4). Out of them, acetyl-CoA acetyltransferase, thioesterase, and glucose-6-phosphate dehydrogenase encoded by *phaA*, *tesB*, and *zwf*, respectively, were accumulated as a major protein in each KM-1 transformant (Fig. 2D).

### Gene disruption in KM-1

**Fig. 3:**
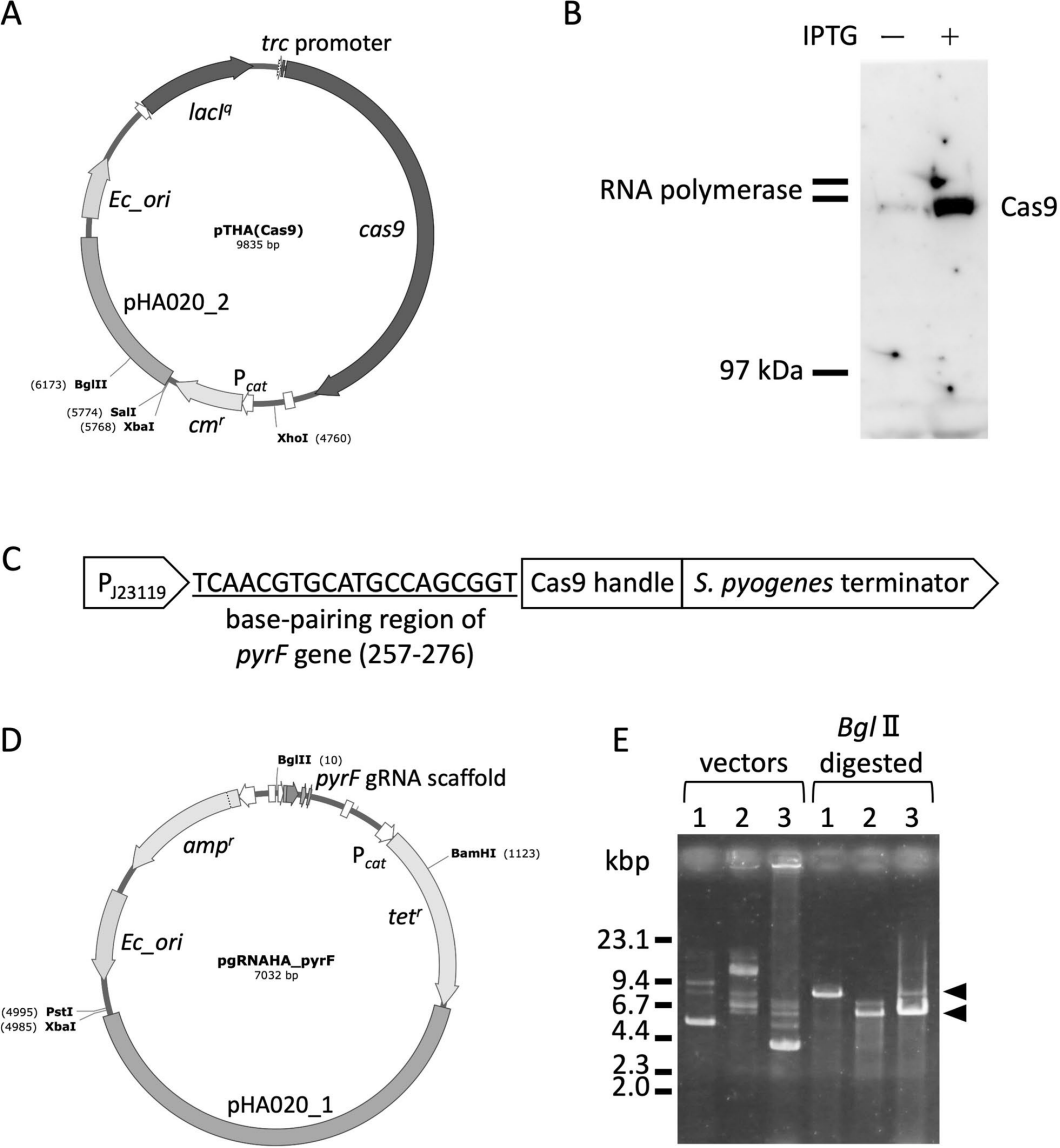
Gene disruption using CRISPR-Cas9 system. **A** pTHA(Cas9) vector was constructed using *S. pyogenes cas9* gene, pTrc99a, and pUCpHAw. Expression of *cas9* was under *trc* promoter regulated by *lacI*^*q*^ and induced by the addition of IPTG. The detailed flow of the vector construction is shown in Additional file 1: Fig. S5. **B** Cas9 protein in KM-1 was detected by western blotting. Positions of the two largest subunits of KM-1 endogenous RNA polymerase are indicated on the left. **C** Expression cassette of guide RNA was prepared on pgRNA-bacteria. It consisted of an artificial promoter (P_J23119_), base-pairing region of *pyrF* gene, Cas9 handle, and *S. pyogenes* terminator. **D** pgRNAHA_pyrF vector was constructed based on pHA1AT_32 and the pgRNA-bacteria including the base-pairing region of *pyrF*. The detailed flow of construction is shown in Additional file 1: Fig. S6. **E** Agarose gel electrophoresis was conducted with linearized plasmid vectors extracted from KM-1 with pTHA(Cas9) (lane 1), *E. coli* with pgRNAHA_pyrF (lane 2), and KM-1 with pTHA(Cas9) and pgRNAHA_pyrF (lane 3). BglII digestion was conducted to linearize the vectors (right half). Two triangles indicate the positions of linearized vectors

Gene disruption of *Halomonas* using homologous recombination combined with CRISPR-Cas9 has been previously reported [11]. Herein, we constructed a pTHA(Cas9) vector with the pUCpHAw and *S. pyogenes cas9* gene under the *trc* promoter to develop a gene disruption system using CRISPR-Cas9 in KM-1 (Fig. 3A, Additional file 1: Fig. S5). The transformation efficiency was much lower than that of pUCpHAw, but it was at a practical level for use (Additional file 1: Table S2). Cas9 protein accumulation in KM-1 cells was detected by western blotting (Fig. 3B). KM-1 was found to be susceptible to 5-FOA at a concentration of 0.5 mg/mL or higher, and we could isolate a mutant of KM-1 exhibiting uracil-dependent and 5-FOA resistance using UV irradiation in our laboratory for another purpose (data not shown). Thus, the *pyrF* gene was chosen as a control for gene disruption and the pgRNAHA_pyrF vector was constructed to disrupt the *pyrF* gene (Fig. 3CD, Additional file 1: Fig. S6). The efficiencies of the secondary transformation of KM-1, including the pTHA(Cas9) vector, were significantly low, but transformants of KM-1 harboring both pTHA(Cas9) and pgRNAHA_pyrF were established (Additional file 1: Table S2). The coexistence of the two vectors was confirmed by plasmid preparation from KM-1 transformants and PCR (Fig. 3E).

**Fig. 4:**
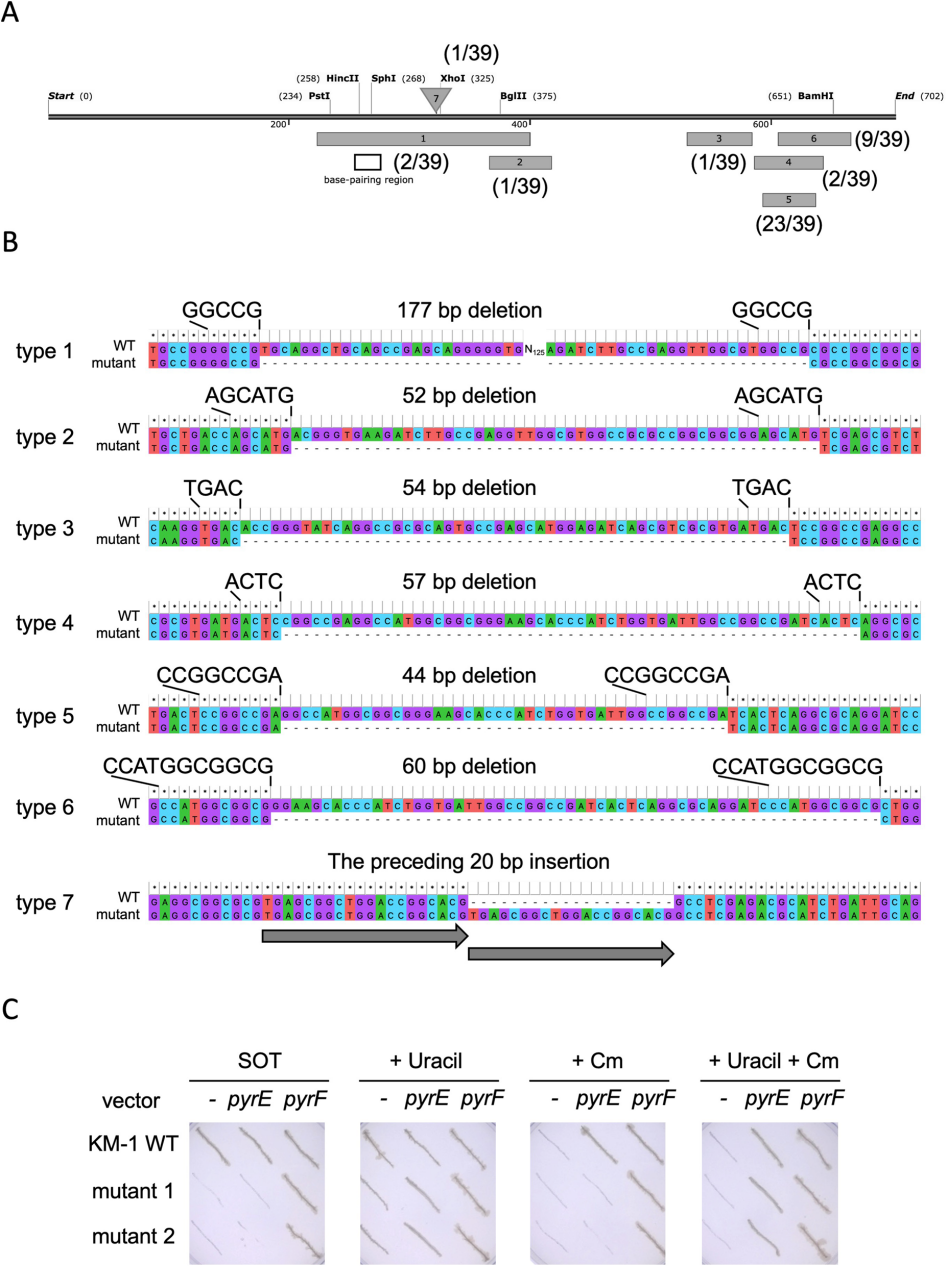
*pyrF* gene disruption and complementation. **A** Locations of mutations in the *pyrF* gene were schematically indicated. Double line, six gray bars numbered from 1 to 6, and a triangle above the double line show coding region of *pyrF*, deletion areas of six types of deletions, and one insertion position, respectively. Numbers in brackets indicate amounts of isolates out of 39 mutants. The base-pairing region of the guide RNA is indicated with an empty box. **B** DNA sequences and microhomologies of each type of mutation are shown. The numbers on the left correspond to seven types of mutations in panel A. **C** KM-1 wild type (KM-1 WT) and *ΔpyrF* mutants (mutant 1 and mutant 2) from type five were transformed with vectors expressing *pyrE* and *pyrF* genes (the sequence IDs are WP_026037794.1 and WP_010629006.1, respectively). KM-1 wild type and *ΔpyrF* mutants were indicated with (−). All strains were grown at 33 ℃ for 48 h on SOT plates containing 3% sucrose supplemented with or without 3 µg/mL chloramphenicol (+ Cm) and 10 µg/mL uracil (+ Uracil)

A strain carrying the two vectors, pTHA(Cas9) and pgRNAHA_pyrF, was subjected to gene disruption. As a result, 45 mutants exhibiting 5FOA-resistant were isolated, and 39 out of the mutants were confirmed to contain deletions in the *pyrF* gene, except one with insertion (Fig. 4A, B). Whereas no mutant was obtained from a strain harboring pTHA(Cas9) and pgRNAHA despite the approximately one-eighth effort of whole mutant-selections (Additional file 1: Table S4). It statistically implies that the DNA disruption depends on the Cas9 and guide RNA for the *pyrF* gene, but not on the random off-target mutation. The mutant phenotypes, 5-FOA resistant and uracil auxotrophy, were complemented by transformation with pCmHAw_Phcp_pyrF but not pCmHAw_Phcp_pyrE (Fig. 4C).

## Discussion

A variety of genetic tools have been developed for gene transduction in *Halomonas*. Conjugation has been widely used for plasmid-based transformation of *Halomonas* using the broad-host-range vector pBBR1MCS [21] and a *Halomonas* native plasmid [22]. An electroporation method has been reported using pBBR1MCS [10, 23]. However, the *Halomonas* sp. KM-1, which is a bacterium with potential in the field of biochemical production, was not effectively transformed by electroporation using pBBR1MCS. Thus, the two plasmids identified in the *Halomonas* sp. A020 [18] were modified and developed into two independent vectors for KM-1 at the practical transformation level. However, many challenges remain, such as the low efficiency of transformation with a vector containing a long gene, such as *cas9*, and one of the secondary transformations was drastically low with a second vector to a KM-1 transformant. Moreover, most *Halomonas* strains, which were previously isolated in our laboratory, were not transformable except one, *Halomonas* sp. A031. Recently, Wang et al. reported that disruption of the *lpxL* gene, which enhanced membrane permeability, allowed *H. bluephagenesis* TD01 to be transformed using an electroporation method [23]. Membrane modification could be a potential alternative to overcome the incompetency or increase the transformation efficiency of *Halomonas* bacteria as well as the use of the overcome classical restriction (ocr) protein, which protects non-methylated DNA against Type I R/M systems [24] and disruption of *recA* for stabilization of heterologous genes [25].

Endogenous, heterologous, and artificial promoters have been adapted to express genes and regulate gene expression in *Halomonas*. For example, a native putative promoter in pHE1 of *H. elongata* exhibited *Pseudomonas syringae inaZ* gene expression in other moderately halophilic bacteria, including four *Halomonas* strains [22]. By randomizing the promoter sequence of a porin gene, a constitutive promoter library was established with a 310-fold variation in transcriptional activity in *H. bluephagenesis* TD01 [26]. Moreover, an inducible promoter was built with a > 200-fold induction by integrating the lac operator into a core promoter region of the porin gene [26]. In this study, an endogenous promoter of the *hcp* gene exhibited constitutive expression of endogenous (such as *phaA* and *pyrF*) and heterologous EGFP genes, and the inducible promoter in *E. coli*, the *trc* promoter, and *lacI*^*q*^, were functional to induce EGFP and *cas9* gene expression by IPTG addition in KM-1 as well. To the best of our knowledge, all promoters of *E. coli* are adaptable to gene expression in *Halomonas*, such as upstream sequences of *cm*^*r*^, *tet*^*r*^, and *lacI* genes. Usage of the *hcp* promoter may be advantageous for economical overexpression and protein purification in *Halomonas*, as well as for biochemical production. To establish *Halomonas* strains as platform bacteria for bioproduction, clarification of endogenous promoters is necessary, which are inducible under conditions such as different temperatures and concentrations of salt and oxygen [27, 28].

The Hcp of KM-1 was identified to be highly accumulated in cells at an exponentially growing phase based on the 2D-proteome analysis in this study, and there are two *hcp* genes, which encode an identical amino acid sequence, in the KM-1 genome. While the porin protein was found to be highly accumulated in *H. bluephagenesis* HD01 [26], porin proteins were not remarkably accumulated in KM-1. The Hcp polymer constructs a puncturing device for the bacterial type VI secretion system (T6SS) to deliver a variety of antibacterial or antieukaryotic effectors into competing microbes and host cells [29, 30]. Combined with duplication of the *hcp* gene and high accumulation of the gene product, Hcp in KM-1 might have some other roles such as that of a chaperone or transporter for general materials rather than a transporter of effectors in the T6SS [31].

Gene disruption systems have also been reported in *Halomonas* spp. Random mutagenesis using transposon Tn*5* was performed to build mutant libraries [32, 33]. Gene-targeted disruption systems were established using two different technologies. One of them was based on vectors with restriction enzyme genes and their recognition sequences, which facilitated gene disruption and allelic exchange by homologous recombination [34–36]. The CRISPR-Cas9 technique was adapted for gene knockdown using CRISPPRi [37] and gene disruption combined with homologous recombination [11, 12]. For gene disruption and allelic exchange, *pyrEF* genes in the uracil synthesis pathway were shown to be useful in *Halomonas*, similar to the yeast system [14]. In this study, the *pyrF* gene was targeted and successfully disrupted using the CRISPR-Cas9 system. The mutations were expected to occur at the target site in the guide RNA by non-homologous DNA end joining (NHEJ) [38]. However, most of the mutation regions did not include the *pyrF* target site. Instead of off-target mutations, the boundary sequences of all deletion mutations contained ruleless microhomologies. In *Zymomonas mobilis*, genomic DNA damage caused by a subtype I-F CRISPR-Cas system was repaired through microhomology-mediated end joining (MMEJ) [39, 40]. A double-stranded DNA break has also been reported to stimulate DNA tandem repeat instability and facilitate mutations [41]. Thus, the off-target mutations appear to be attributed to MMEJ repair in direct repeats.

## Supplementary Information

**Additional file 1.** **Table S1**. List of primers used in this work. **Table S2.** Comparison of transformation efficiencies of different strains of *Halomonas*. **Table S3.** N-terminal amino acid sequences of highly accumulated proteins. **Table S4.** Results of *pyrF* gene disruption using the CRISPR-Cas9 system. **Fig. S1.** Construction of a shuttle vector, pUCpHAw. The first *Escherichia coli-Halomonas* shuttle vector was constructed using pUC19 and the small *Halomonas* sp. A020 plasmid, pHA020_2, with a chloramphenicol-resistant gene (*cm*^*r*^/*cat*). **Fig. S2.** Construction of pHA1AT_32. The second shuttle vector was constructed from the pUCpHAw by substitution of the pHA020_2 region and *cm*^*r*^/*cat* with an origin region of the large *Halomonas* sp. A020 plasmid, pHA020_1, and a tetracycline-resistant gene (*tet*), respectively. **Fig. S3.** Construction of EGFP expression vectors. EGFP gene was cloned into the pUCpHAw, resulting in pUCpHAw_EGFP. Three promoter regions, upstream regions of the highly expressed genes, *hcp* and *phasin*, and *trc* promoter with *lacI*^*q*^, were cloned into the 5’ flanking region of the EGFP gene in pUCpHAw_EGFP. **Fig. S4.** Construction of gene expression vectors. The ampicillin-resistant gene (*amp*^*r*^) was removed from the pUCpHAw_Phcp_EGFP, and the EGFP gene was substituted with KM-1 genes, such as *zwf*, *phaA*, and *tesB*. **Fig. S5.** Construction of pTHA(Cas9). After cloning of *Streptococcus pyogenes cas9* gene in the pTrc99a vector, a DNA fragment containing *lacI*^*q*^, *trc* promoter, and *cas9* was cloned into the pUCpHAw, resulting in pTHA(Cas9). **Fig. S6.** Construction of guide RNA expression vectors. pgRNA-bacteria_pyrF vector was constructed by cloning a 20-base DNA fragment in *pyrF* to an adjacent site of the PAM sequence of the pgRNA-bacteria vector. pgRNAHA_pyrF vector was constructed by fusion of the pgRNA-bacteria_pyrF and a part of pHA1AT_32. **Fig. S7.** Complementation of *pyrF* gene in *ΔpyrF* mutants. Expression vectors, pCmHAw_Phcp_pyrF and pCmHAw_Phcp_pyrE, were constructed by replacement of the EGFP gene in pCmHAw_Phcp_EGFP with KM-1 *pyrF* and *pyrE* genes, respectively. **Fig. S8.** Multiple sequence alignments of Phasin and Hcp. Multiple sequence alignments of *Halomonas* Phasin (A) and Hcp (B) were performed using ClustalW ver. 2.1. **Fig. S9.** DNA electrophoresis of *pyrF* gene disruption mutants. Genomic DNA regions including the *pyrF* gene of KM-1 wild-type and mutants were amplified and analyzed using the agarose gel electrophoresis.

**Additional file 2.** DNA sequence and information of the vector, pUCpHAw.

**Additional file 3.** DNA sequence and information of the vector, pHA1AT_32.

**Additional file 4.** DNA sequence and information of the vector, pUCpHAw_EGFP.

**Additional file 5.** DNA sequence and information of the vector, pgRNAHA_pyrF.

**Additional file 6.** DNA sequence and information of the vector, pTHA(Cas9).
