# Supplementary material for: Establishment of genetic tools for genomic DNA engineering of Halomonas sp. KM-1, a bacterium with potential for biochemical production
Source: Microb Cell Fact. 2022 Jun 20;21:122. doi: 10.1186/s12934-022-01797-2 (PMC9208146; doi:10.1186/s12934-022-01797-2)
Supplement: Supplementary file 1 — Additional file 1. Table S1. List of primers used in this work. Table S2. Comparison of transformation efficiencies of different strains of Halomonas. Table S3. N-terminal amino acid sequences of highly accumulated proteins. Table S4. Results of pyrF gene disruption using the CRISPR-Cas9 system. Fig. S1. Construction of a shuttle vector, pUCpHAw. The first Escherichia coli-Halomonas shuttle vector was constructed using pUC19 and the small Halomonas sp. A020 plasmid, pHA020_2, with a chloramphenicol-resistant gene (cmr/cat). Fig. S2. Construction of pHA1AT_32. The second shuttle vector was constructed from the pUCpHAw by substitution of the pHA020_2 region and cmr/cat with an origin region of the large Halomonas sp. A020 plasmid, pHA020_1, and a tetracycline-resistant gene (tet), respectively. Fig. S3. Construction of EGFP expression vectors. EGFP gene was cloned into the pUCpHAw, resulting in pUCpHAw_EGFP. Three promoter regions, upstream regions of the highly expressed genes, hcp and phasin, and trc promoter with lacIq, were cloned into the 5’ flanking region of the EGFP gene in pUCpHAw_EGFP. Fig. S4. Construction of gene expression vectors. The ampicillin-resistant gene (ampr) was removed from the pUCpHAw_Phcp_EGFP, and the EGFP gene was substituted with KM-1 genes, such as zwf, phaA, and tesB. Fig. S5. Construction of pTHA(Cas9). After cloning of Streptococcus pyogenes cas9 gene in the pTrc99a vector, a DNA fragment containing lacIq, trc promoter, and cas9 was cloned into the pUCpHAw, resulting in pTHA(Cas9). Fig. S6. Construction of guide RNA expression vectors. pgRNA-bacteria_pyrF vector was constructed by cloning a 20-base DNA fragment in pyrF to an adjacent site of the PAM sequence of the pgRNA-bacteria vector. pgRNAHA_pyrF vector was constructed by fusion of the pgRNA-bacteria_pyrF and a part of pHA1AT_32. Fig. S7. Complementation of pyrF gene in ΔpyrF mutants. Expression vectors, pCmHAw_Phcp_pyrF and pCmHAw_Phcp_pyrE, were constructed by replacement of the [file 12934_2022_1797_MOESM1_ESM.docx]

| **Additional file 1: Table S1. List of primers** | |
| --- | --- |
| Primer | Sequence (5'→3') |
| AT031 | CCTCTAGAGTCGACCGATCCCCGGGTACCGGTCGC |
| AT044 | GCATCAAGGAACTCCTTGGAATTCTAGAGTCGCGG |
| AT097 | ATGGTGAGCAAGGGCGAGGAGCTGTTCACC |
| AT098 | GGGGATCCTCTAGAGGATCGACTTCGACCTCTTG |
| AT099 | GCCCTTGCTCACCATGGTCTGCTCCTTGACGATG |
| AT141 | GCCCTTGCTCACCATGGTCTGTTTCCTGTGTG |
| AT142 | GGGGATCCTCTAGAGTGGTGCAAAACCTTTCGC |
| AT143 | GGGGATCCTCTAGAGGGTCGCTCTAGGGAGTGC |
| AT144 | GCCCTTGCTCACCATGAGACACCTCCTACGTTG |
| AT170 | TCGGTGAACTGGATCTGAATTCGAGCTCGGTACCC |
| AT171 | ACTCGAGTAAGGATCATGATTACGCCAAGCTCGAG |
| MM05 | CGAGCTCTTGAGATCCTTTTTTTCTGCG |
| MM06 | GATCTCAAGAGCTCGGTACCCAGAAGCC |
| MM12 | AAGGAACTTCAGGTCGAGGTGGCCCG |
| MM21 | TACGCCAAGCTCGAGATTCCTGTAGAAATTAGCGG |
| MM22 | TACGCCAAGCTCGAGGCTTCCGGCCCTTTTCTCTA |
| MM24 | TAGAGTCGACCCGGGCCTACCGCTGTTTGGCTTCG |
| MM26 | CCCGGGTCGACTCTAGAGGATCCC |
| MM27 | CTCGAGCTTGGCGTAATCATGGTC |
| MM28 | GACCTGAAGTTCCTTCCTACCGCTGTTTGGCTTCG |
| MM29 | GATTTCATTTTAGCTTCCTTAGCTCCTG |
| MM31 | AAGCTAAAATGAAATCTAACAATGCGCTCA |
| MM54 | AGTTCCTTGATGCCGACGTC |
| MM55 | ATGGTGATGATGATGGTGGTGCATGGTCTGCTCCTTGACGATG |
| MM60 | CATCATCATCACCATATGAAGGCAACCAGGCGGTC |
| MM61 | CGGCATCAAGGAACTTCAGTAGTCCTCGTACCAGC |
| MM62 | CATCATCATCACCATATGCGTCTCGATTCCGTCGT |
| MM63 | CGGCATCAAGGAACTCTACTTCTCCAGCAACAGCG |
| MM70 | CATCATCATCACCATATGTCCGACGCCCTGAACCA |
| MM71 | CGGCATCAAGGAACTTTAATCTCCGTCGTGTAGCC |
| MM80 | CATCATCATCACCATGTGTCCTGTGATTCACCGCT |
| MM81 | CGGCATCAAGGAACTTCAGCTTACCGCCAGCTCCT |
| MM82 | CGGGCTGGTATAATCGCCGC |
| MM83 | CCCGGTACCTTAGCTTGCCG |
| MM84 | CATCATCATCACCATATGCAAGCCTATCAGCGGGA |
| MM85 | CGGCATCAAGGAACTTCACTCGGCGGCGACACCGT |
| YA_cas9a | AGGAAACAGACCATGGATAAGAAATACTCAATAGG |
| YA_cas9b | CAAAACAGCCAAGCTTTAGTCACCTCCTAGCTGAC |
| YA_cas9c | GGCTTCTGGGTACCGCTCGAGTGCTTAATTTGATGCCTGGC |
| YA_cas9d | ATTGCCGCACAAGCTATCGATCTGTCAGACCAAGTTTACTC |
| YA_pChl1 | TCGAGCTCGGTACCCAGAAGCCACTGGAGCACCTC |
| YA_pChl2 | CTCTAGAGGATCCCCACCAGGCGTTTAAGGGCACC |
| YA_pHA3_3 | GAGTCGACCTGCAGGAGTTCCTTGATGCCGACGTC |
| YA_pHA3_4 | ATTACGCCAAGCTTGTGCGGCAATGGTCACAGTGC |
| YT01 | CAACGTGCATGCCAGCGGTGTTTTAGAGCTAGAAA |
| YT02 | CGCTGGCATGCACGTTGAACTAGTATTATACCTAG |

| **Additional file 1: Table S2. Transformation efficiency of *Halomonas* strains** | | | |  |
| --- | --- | --- | --- | --- |
| host | vector | colony formation / µg of DNA | Reference | |
| O-1 | pBBR1MCS-5 (*gen^r^*) | 10^4^ | Harris *et al*. (2016) [1] | |
|  | pBBR1MCS (*cm^r^*) | 10^3^ | Harris *et al*. (2016) [1] | |
|  | pBBR1MCS-3 (*tet^r^*) | Not obtained | Harris *et al*. (2016) [1] | |
| KM-1 | pBBR1MCS (*cm^r^*) | 40 | This work | |
|  | pUCpHAw (*cm^r^*) | 10^2-3^ | This work | |
|  | pHA1AT_32 (*tet^r^*) | 10^3^ | This work | |
|  | pTHA(Cas9) | 4 | This work | |
| KM-1 + pTHA(Cas9) | pHA1AT_32 | 10^-1^ | This work | |
|  | pgRNAHA | 10^-1^ | This work | |
|  | pgRNAHA_pyrF | 10^-1^ | This work | |

| **Additional file 1: Table S3. N-terminal amino acid sequences and predicted proteins** | | | |
| --- | --- | --- | --- |
| N-terminal amino acid sequence* | pI | kDa | Annotation of predicted protein |
| PTP-YI (PTPCYI) | 5 | 20 | hemolysin coregulated protein (Hcp) family type Ⅵ secretion system effector |
| SKTQDK | 7 | 14 | phasin (polyhydroxyalkanoate-associated protein) |
| *N-terminal amino acid sequences were determined by Edman degradation. | | | |

| **Additional file 1: Table S4. Results of screening of gene disruption mutants.** | | | | |  |
| --- | --- | --- | --- | --- | --- |
| target gene | 1st screening | 2nd screening | subjected to DNA sequencing | | |
|  |  |  | *ΔpyrF* | *ΔpyrE* | unknown |
| mock | 200 | 0 | 0 | 0 | 0 |
| *pyrF* | 1624 | 45 | 39 | 2 | 4 |


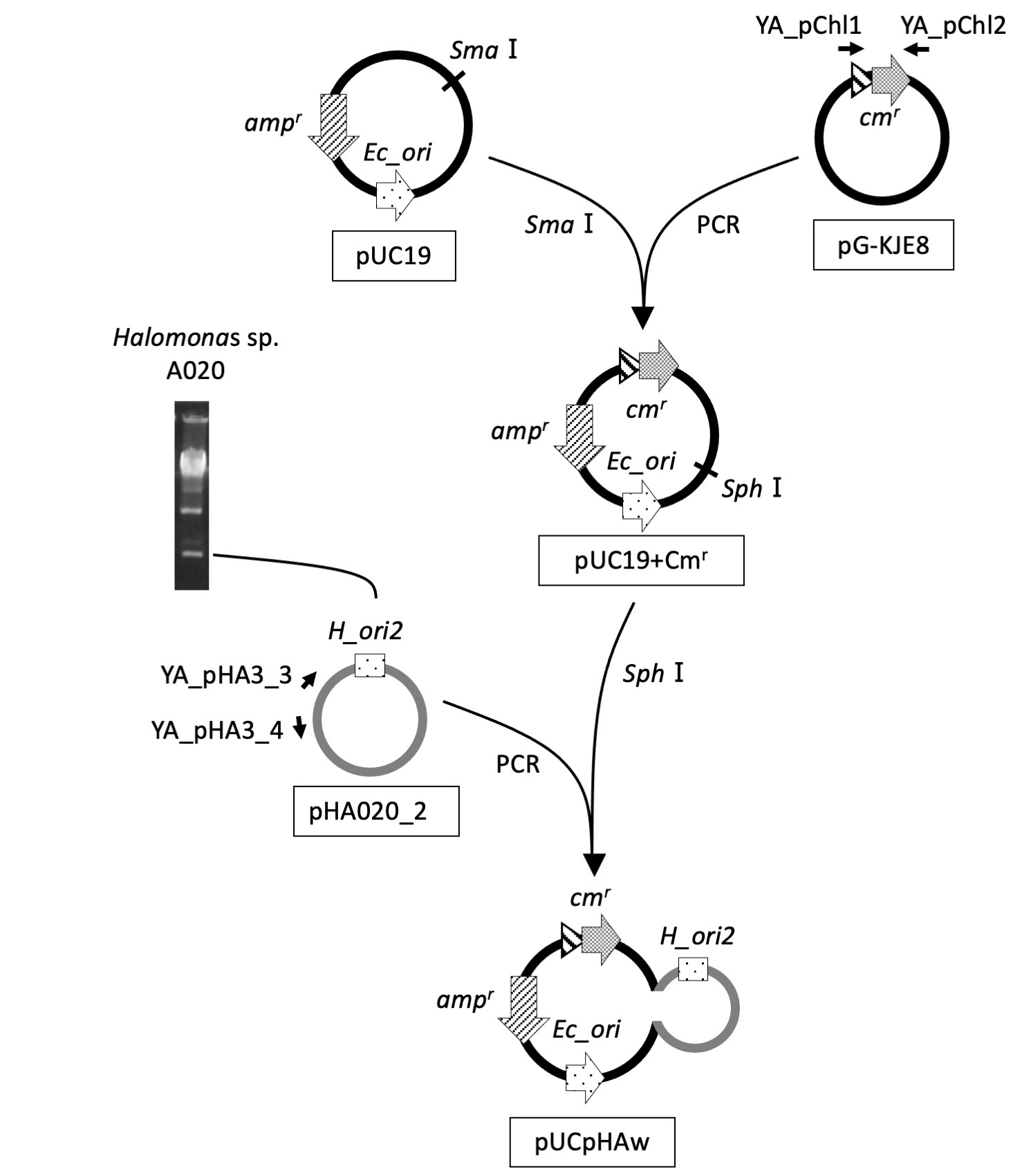


**Additional file 1: Fig. S1. Construction of a shuttle vector, pUCpHAw**

A shuttle vector, pUCpHAw, was constructed using pUC19, *cm^r^* from pG-KJE8, and pHA020_2. PCR amplified *cm^r^* with primers (YA_pChl1 and YA_pChl2) was cloned into a *Sma*Ⅰ site of pUC19, resulting in pUC19+*cm^r^*. A whole pHA020_2 was amplified by PCR with primers (YA_pHA3_3 and YA_pHA3_4) and cloned into an *Sph*Ⅰ site of the pUC19+*cm^r^*, resulting in pUCpHAw.


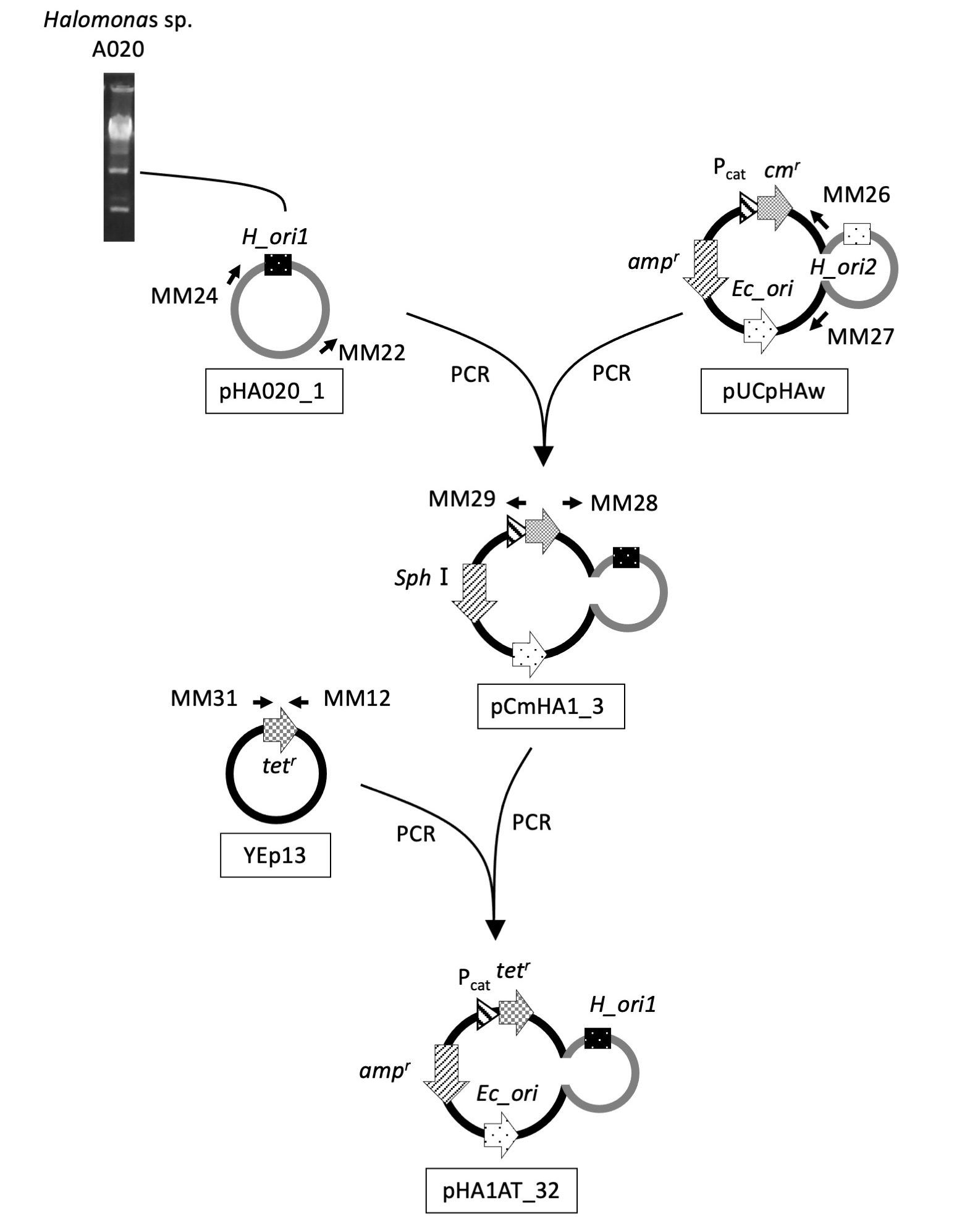


**Additional file 1: Fig. S2. Construction of pHA1AT_32**

A shuttle vector, pHA1AT_32, was constructed from pUCpHAw by substitution of the replication origin and selection marker gene. The pHA020_2 region in pUCpHAw was removed by PCR with primers (MM26 and MM27) and exchanged with an origin region of pHA020_1, which was amplified by PCR with primers (MM22 and MM24). Then *cm^r^* was removed by PCR with primers (MM28 and MM29) and substituted with *tet^r^* of YEp13 amplified by PCR with primers (MM12 and MM31).


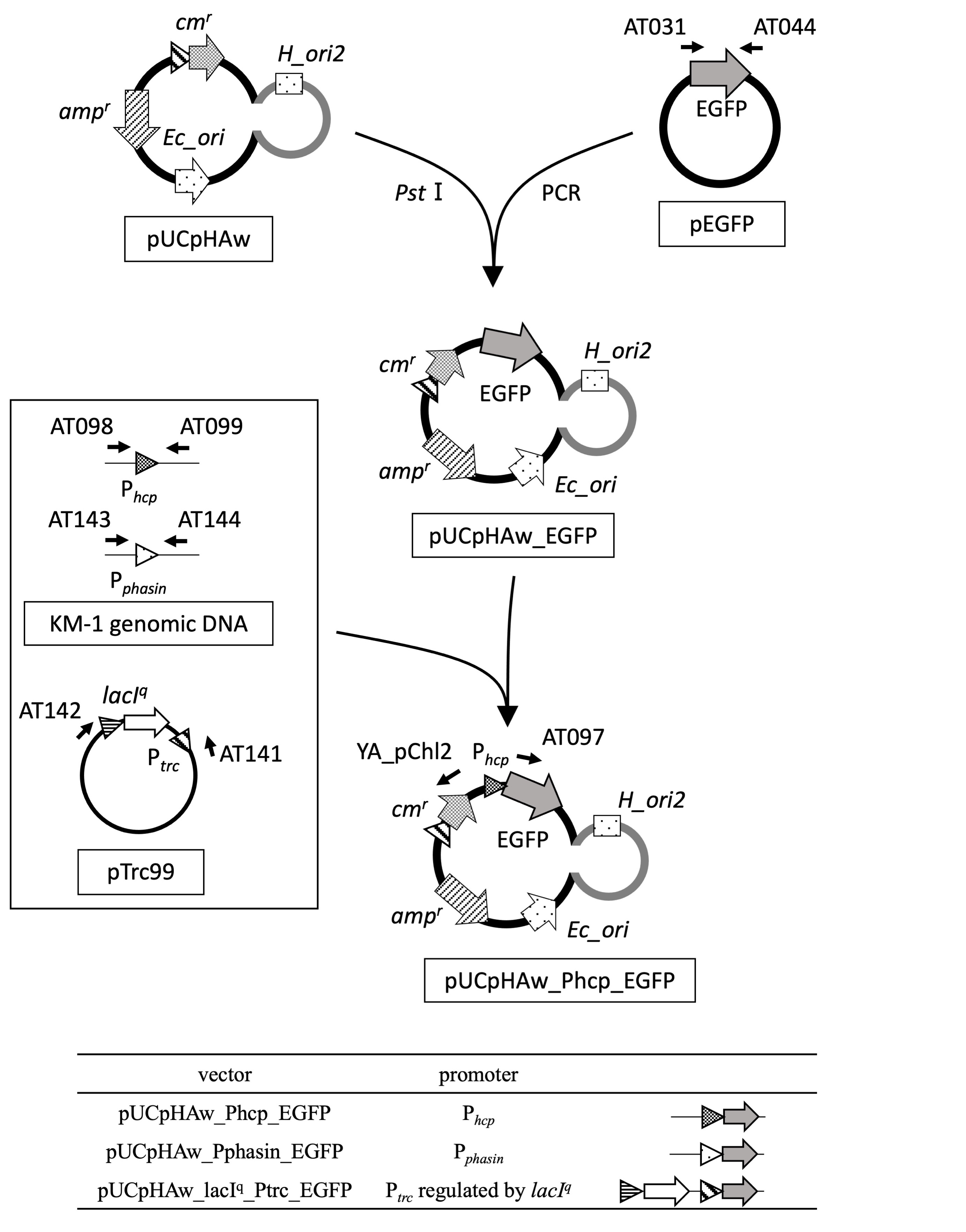


**Additional file 1: Fig. S3. Construction of EGFP expression vectors**

EGFP was amplified by PCR with primers (AT031 and AT044) and cloned into a *Pst*Ⅰ site of pUCpHAw, resulting in pUCpHAw_EGFP. Three promoter regions, upstream regions of *hcp* and *phasin* genes of KM-1, and *trc* promoter with *lacI^q^* of pTrc99a vector, were amplified by PCR using primers (AT098 and AT099), (AT143, AT144), and (AT141 and AT142), respectively. Those DNA fragments were assembled with a DNA fragment of pUCpHAw_EGFP, which was amplified by PCR with primers (YA_pChl2 and AT097), resulting in three vectors, pUCpHAw_Phcp_EGFP, pUCpHAw_Pphasin_EGFP, and pUCpHAw_lacI^q^_Ptrc_EGFP.


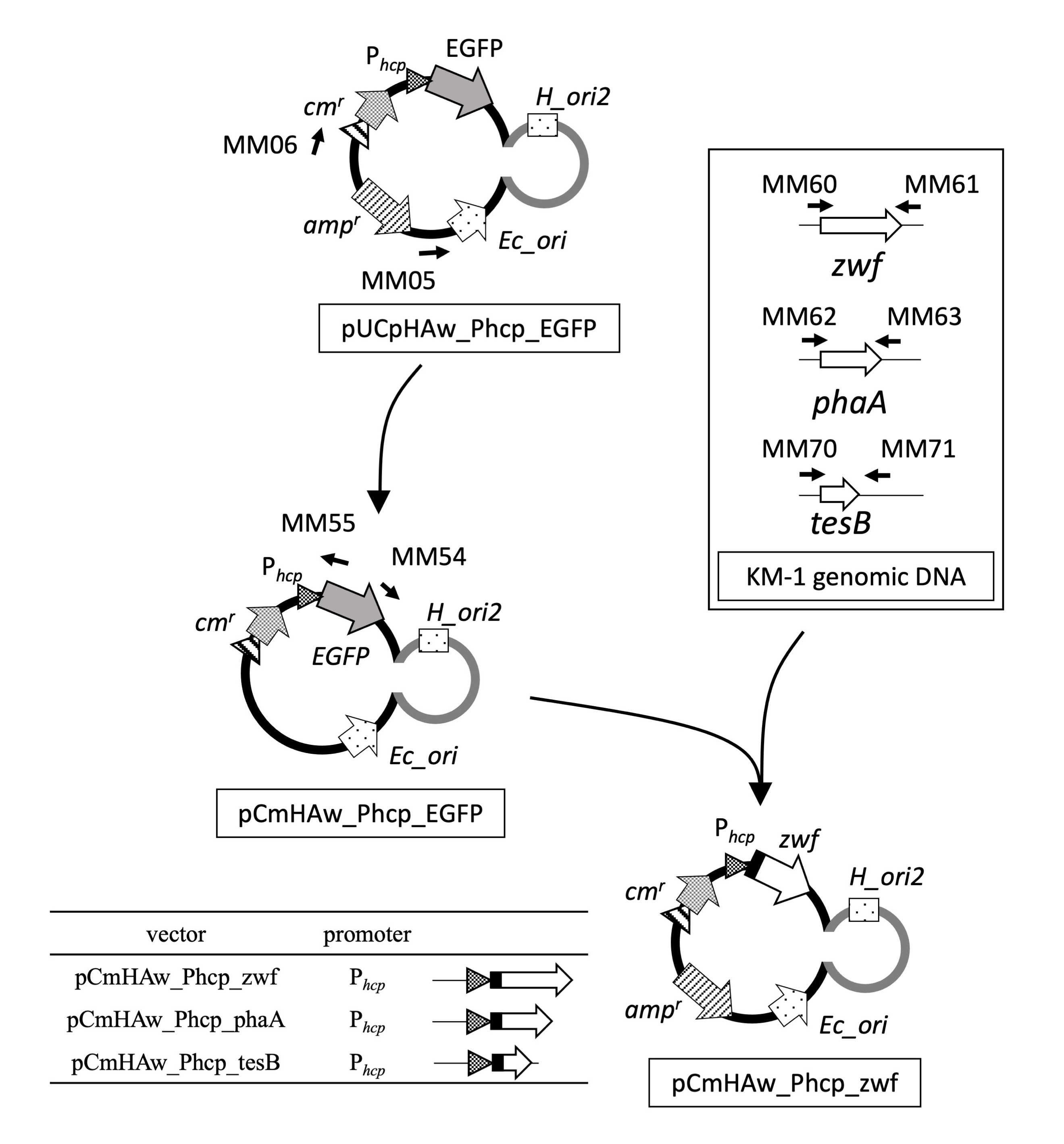


**Additional file 1: Fig. S4. Construction of gene expression vectors**

The pUCpHAw_Phcp_EGFP was amplified by PCR with primers (MM05 and MM06) and assembled to remove *amp^r^*, resulting in pCmHAw_Phcp_EGFP. KM-1 genes, such as *zwf*, *phaA,* and *tesB*, were amplified by PCR with primers (MM60 and MM61), (MM62 and MM63), and (MM70 and MM71), respectively. The 7×His-tag sequence (shown in a black square) was added at the N-terminal end of each gene. The EGFP in pCmHAw_Phcp_EGFP was exchanged with the DNA fragments, resulting in pCmHAw_Phcp_zwf, pCmHAw_Phcp_phaA, and pCmHAw_Phcp_tesB.


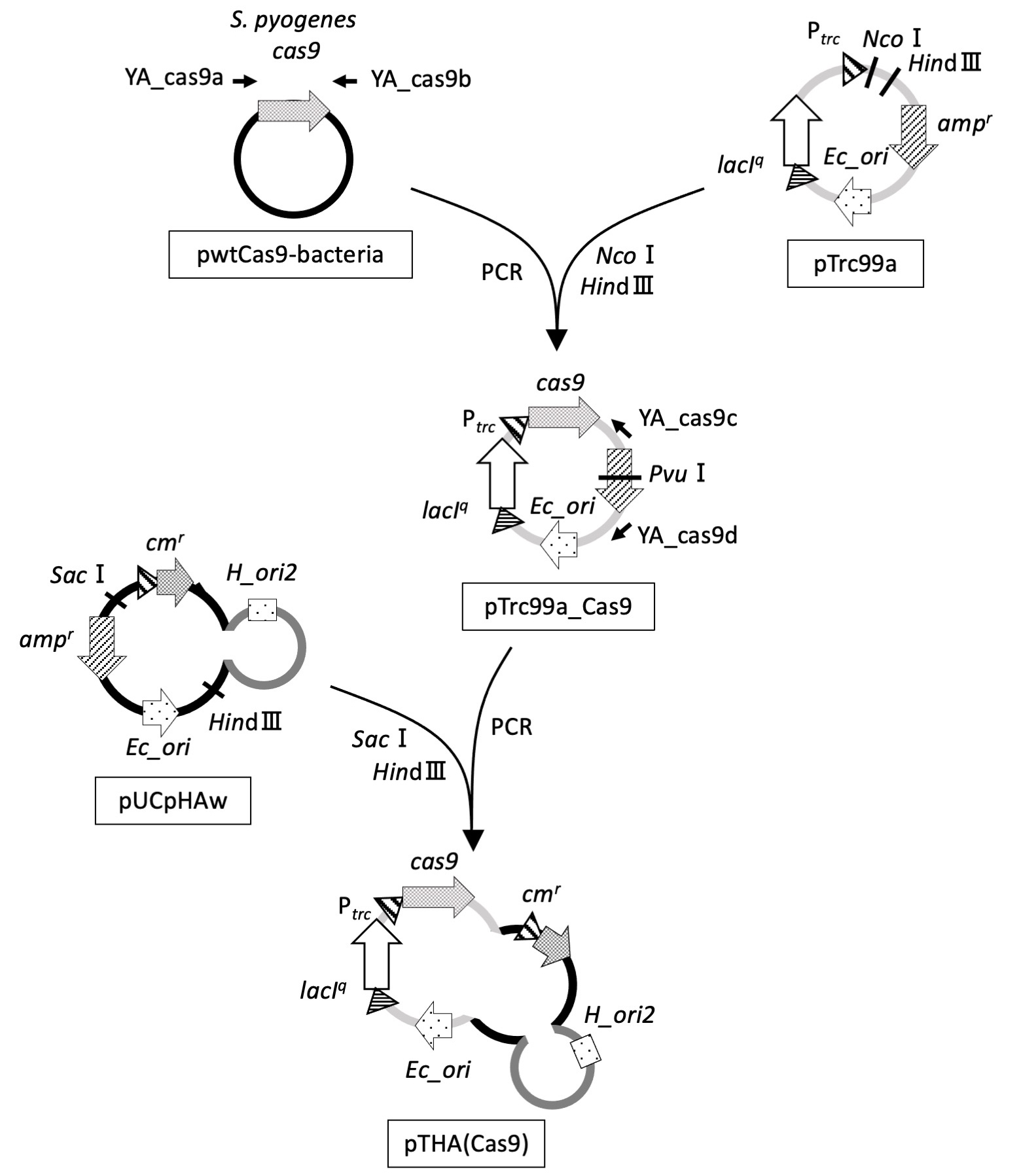


**Additional file 1: Fig. S5. Construction of pTHA(Cas9)**

The *cas9* gene fragment, which was amplified by PCR using pwtCas9-bacteria and primers (YA_cas9a and YA_cas9b), was cloned by In-Fusion at a *Nco*Ⅰ-*Hin*dⅢ site of pTrc99a vector, resulting in pTrc99a_Cas9. A DNA fragment containing *lacI^q^*, *trc* promoter, and *cas9* was amplified by PCR using pTrc99a_Cas9 and primers (YA_cas9c and YA_cas9d) and cloned by In-Fusion at a *Sac*Ⅰ-*Hin*dⅢ site of pUCpHAw, resulting in pTHA(Cas9).


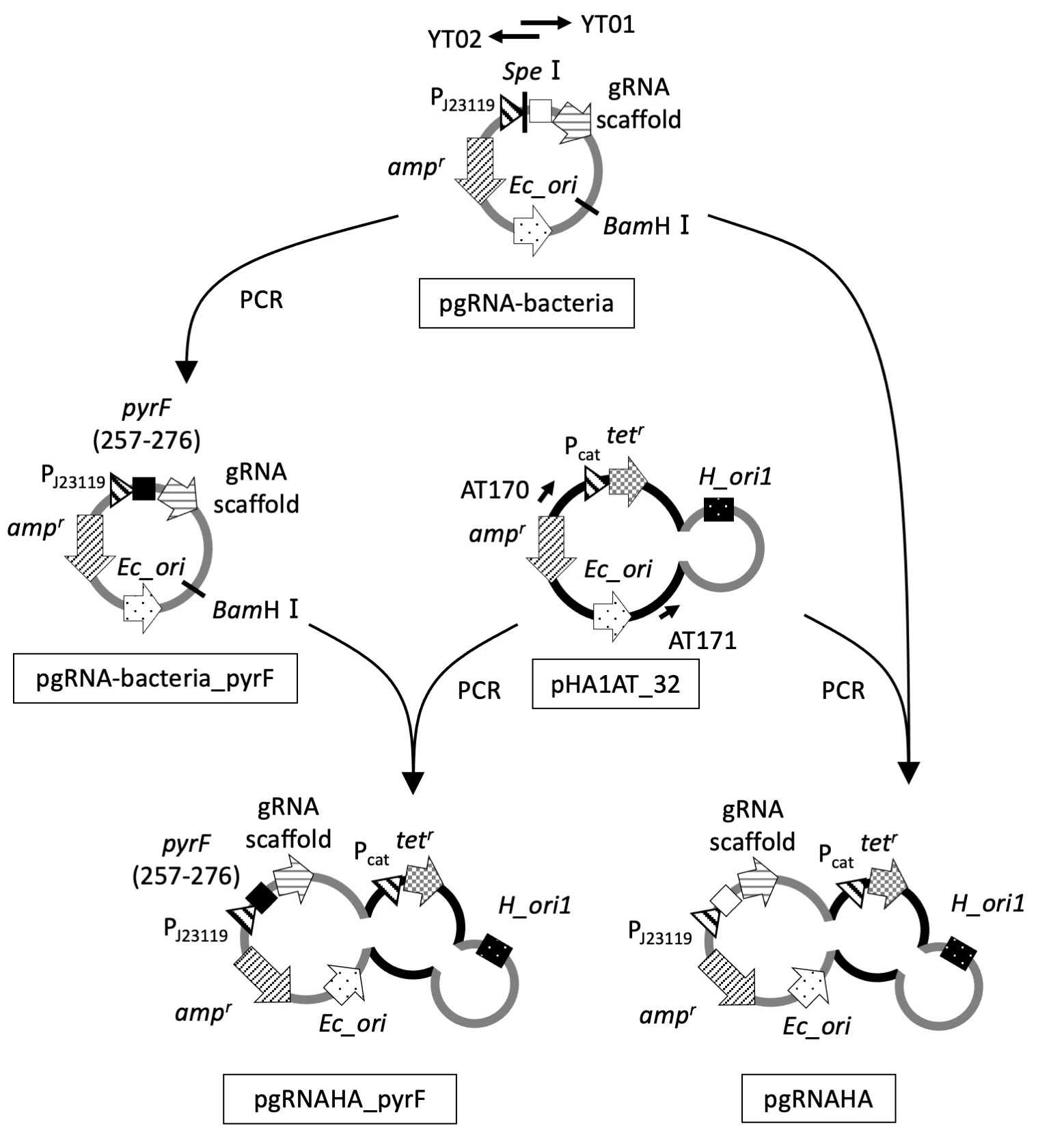


**Additional file 1: Fig. S6. Construction of guide RNA expression vectors**

A 20-base DNA fragment in *pyrF* adjacent to a PAM sequence was cloned into pgRNA-bacteria by PCR with primers (YT01 and YT02), resulting in pgRNA-bacteria_pyrF. After *Bam*HⅠ digestion of pgRNA-bacteria_pyrF, the DNA fragment was cloned by In-Fusion with the pHA1AT_32 fragment, which was amplified by PCR with primers (AT170 and AT171), resulting in pgRNAHA_pyrF. To construct a control vector, pgRNAHA, pgRNA-bacteria was digested with *Bam*HⅠ and the DNA fragment was cloned by In-Fusion with pHA1AT_32 in the same way as gRNAHA_pyrF construction.


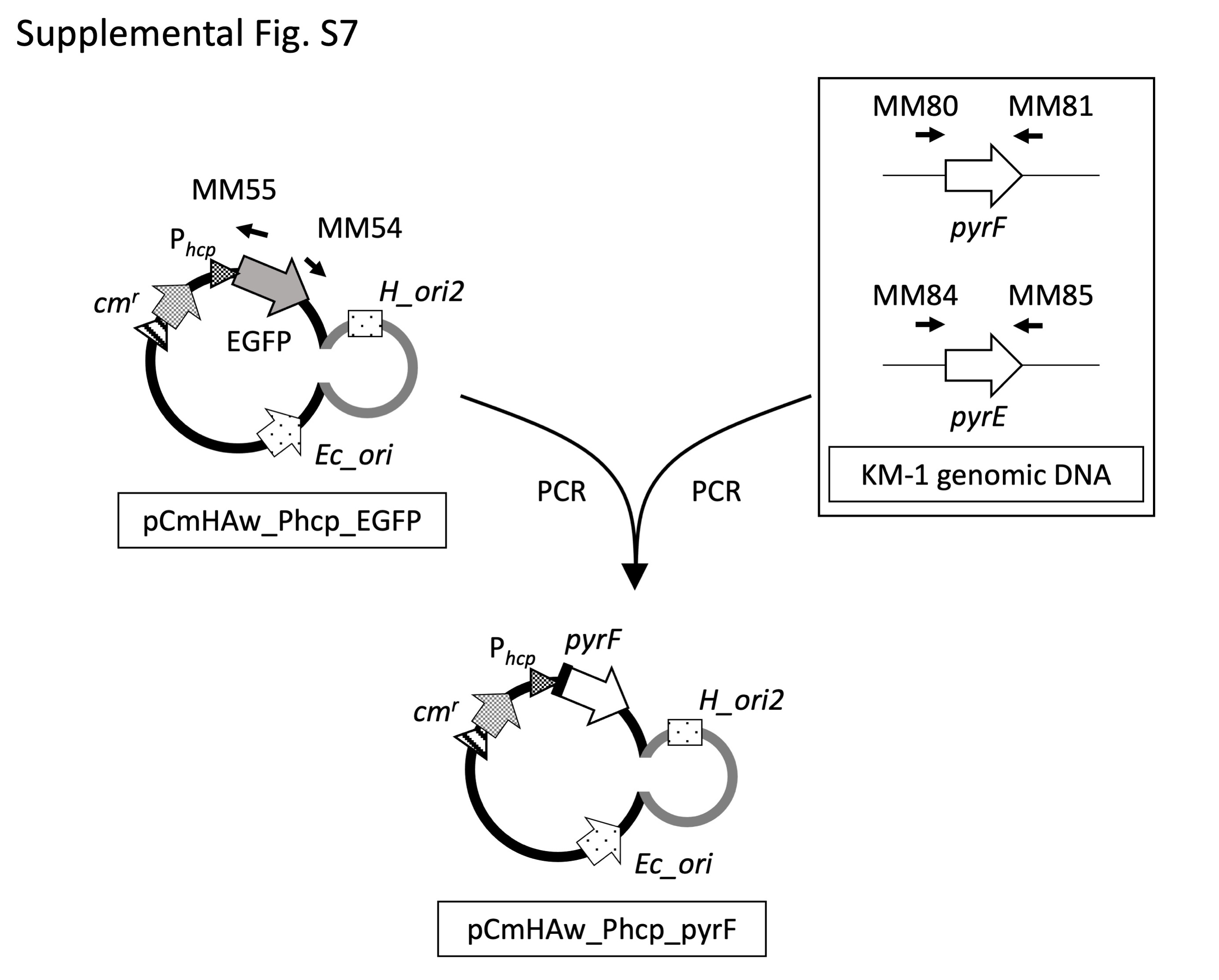


**Additional file 1: Fig. S7. Complementation of *pyrF* gene in *ΔpyrF* mutants**

Expression vectors, pCmHAw_Phcp_pyrF and pCmHAw_Phcp_pyrE, were constructed by replacemant of the EGFP gene in pCmHAw_Phcp_EGFP with KM-1 *pyrF* and *pyrE* genes, respectively in the same way as pCmHAw_Phcp_zwf construction described in Additional file 1: Fig. S5.

**
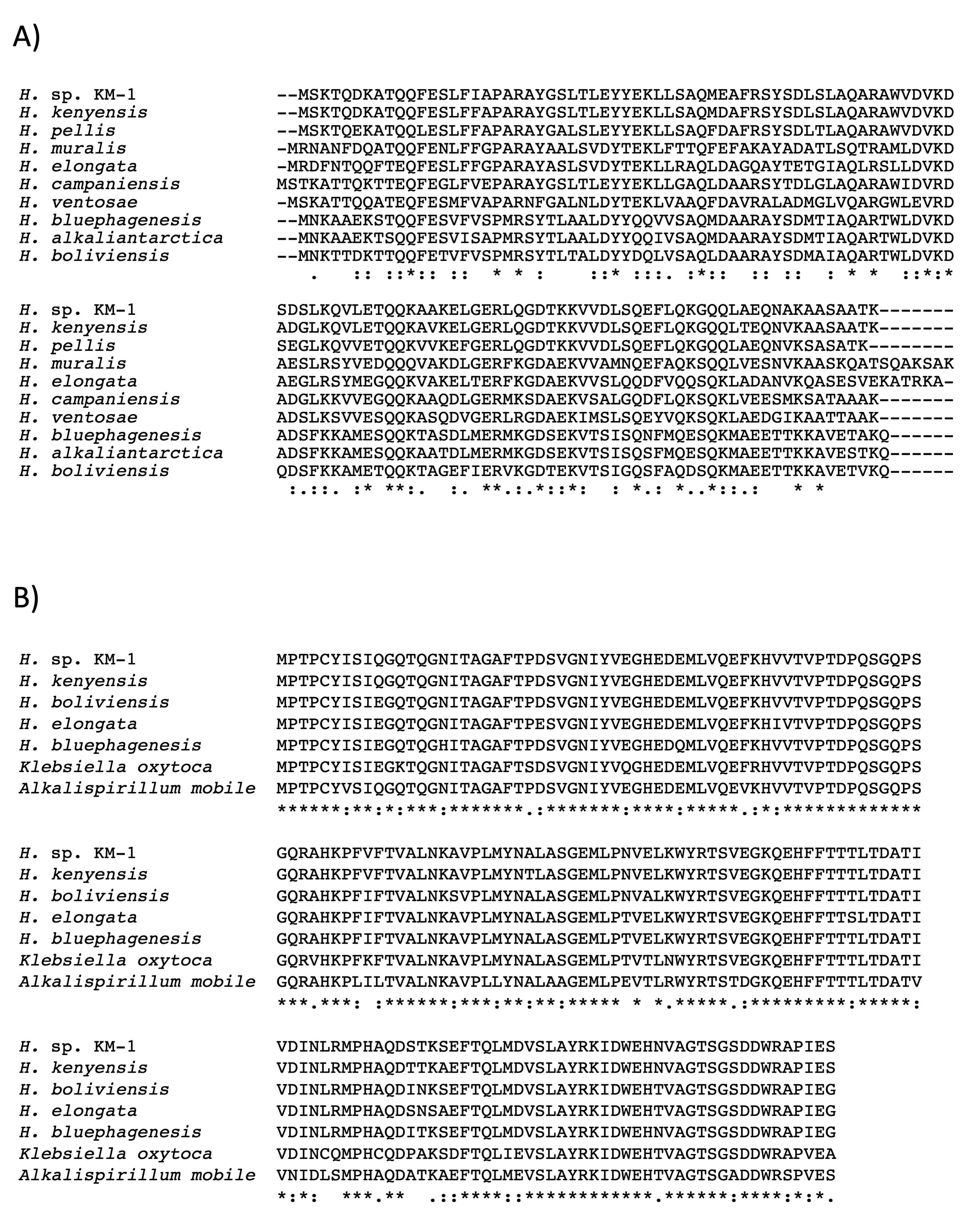
**

**Additional file 1: Fig. S8. Multiple sequence alignments of Phasin and Hcp**

A) Multiple sequence alignment of Phasin proteins was performed using ClustalW ver. 2.1 [2] and 10 Phasin proteins; accession numbers were *Halomonas* sp. KM-1 (LC677174), *Halomonas kenyensis* (WP_181514615), *Halomonas pellis* (WP_149327764), *Halomonas campaniensis* (WP_183329477), *Halomonas ventosae* (WP_106230852), *Halomonas bluephagenesis* (WP_009721314), *Halomonas boliviensis* (WP_040481094), *Halomonas alkaliantarctica* (WP_030073995), *Halomonas muralis* (WP_089729872), and *Halomonas elongata* (WP_013332053).

B) Multiple sequence alignment of Hcp was performed using ClustalW ver. 2.1 [2] and *Halomonas* and Gammaproteobacteria species; accession numbers were *Halomonas* sp. KM-1 (LC677173), *Halomonas kenyensis* (WP_181517154), *Halomonas bluephagenesis* (WP_009722783), *Halomonas boliviensis* (WP_040480043), *Halomonas elongata* (WP_065241715), *Alkalispirillum mobile* (WP_121442090), and *Klebsiella oxytoca* (HAT1682916).


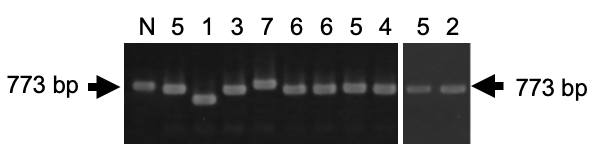


**Additional file 1: Fig. S9. DNA electrophoresis of *pyrF* gene disruption mutants.**

Genomic DNA regions including the *pyrF* gene (shown in Fig. 4A) of KM-1 wild type and mutants were amplified using the primers, MM82 and MM83, and analyzed using the agarose gel electrophoresis. The length of the natural *pyrF* gene was indicated by 773 bp and arrows. Numbers on the top of photos indicate mutation types shown in Fig. 4B, and N shows the natural *pyrF* gene.

**References**

1. Harris JR, Lundgren BR, Grzeskowiak BR, Mizuno K, Nomura CT: **A rapid and efficient electroporation method for transformation of *Halomonas* sp. O-1.** *J Microbiol Methods* 2016, **129:**127-132.

2. Larkin MA, Blackshields G, Brown NP, Chenna R, McGettigan PA, McWilliam H, Valentin F, Wallace IM, Wilm A, Lopez R, et al: **Clustal W and Clustal X version 2.0.** *Bioinformatics* 2007, **23:**2947-2948.
